# Emendation to Rule 8 of the International Code of Nomenclature of Prokaryotes to clarify the meaning of the term ‘stem’

**DOI:** 10.1099/ijsem.0.006833

**Published:** 2025-07-07

**Authors:** Aharon Oren

**Affiliations:** 1 Institute of Life Sciences, The Hebrew University of Jerusalem, The Edmond J. Safra Campus, 9190401, Jerusalem, Israel

**Keywords:** emendation, stem, International Code of Nomenclature of Prokaryotes, International Committee on Systematics of Prokaryotes

## Abstract

Following a proposal to add a Note to Rule 8 of the International Code of Nomenclature of Prokaryotes to clarify the meaning of the term ‘stem’, I hereby report the outcome of the ballot on this proposal by the members of the International Committee on Systematics of Prokaryotes.

## Introduction

In September 2024, A. Oren, M. Kostovski and M.J. Pallen [1] published a proposal to add a Note to Rule 8 of the International Code of Nomenclature of Prokaryotes (ICNP) [2] to clarify the meaning of the term ‘stem’. A public discussion of the proposal was open between 17 September 2024 and 16 March 2025 to comply with Article 13(b) (4) and Article 4(d) of the statutes of the International Committee on Systematics of Prokaryotes (ICSP) [3]. No comments were posted.

The members of the ICSP voted on the proposals in March–June 2025. E.R.B. Moore (Chair, ICSP) and C.M. Manaia (Vice-Chair, ICSP) collated the results of the vote. Out of the 55 members of the ICSP with voting rights, 40 endorsed the emendation, 0 voted against, and there were 2 abstentions. Based on these votes, the following note to Rule 8 was approved:

*Note*. The term ‘stem’, when used in the ICNP, corresponds with that part of the word that does not vary among the forms of the noun in the oblique cases, i.e. cases other than the nominative, and which can be obtained by deleting the ending of the genitive singular.

## Abbreviations

ICNP: International Code of Nomenclature of Prokaryotes
ICSP: International Committee on Systematics of Prokaryotes
